# Gastric varices and splenic vein obstruction during steroid treatment for autoimmune pancreatitis: A case report and literature review

**DOI:** 10.1097/MD.0000000000011940

**Published:** 2018-08-24

**Authors:** Lindsay A. Juarez, Roop R. Gupta, Gregory W. Ruhnke

**Affiliations:** a Saint James School of Medicine, Park Ridge; b Gastroenterology, Department of Internal Medicine, Mercy Hospital and Medical Center; c Section of Hospital Medicine, Department of Medicine, University of Chicago, Chicago, IL.

**Keywords:** autoimmune pancreatitis, gastric varices, sinistral portal hypertension, splenic vein thrombosis, steroid therapy

## Abstract

**Rationale:**

Few cases of autoimmune pancreatitis (AIP) complicated by gastric varices, in the absence of splenic vein obstruction, have been described in the medical literature. The findings in this case parallel those of 3 previously described cases from Japan and support a pathologic explanation for the evolution of gastric varices in relation to early splenomegaly and the role of steroid therapy for AIP.

**Patient concerns:**

A 50-year-old male with a history of transfusion-requiring erosive gastritis and recently diagnosed AIP on steroid therapy for 2 weeks presented with a 2-day history of lightheadedness, abdominal pain, and melena.

**Diagnosis:**

Esophagogastroduodenoscopy (EGD) revealed prominent varices in the gastric fundus. An abdominal ultrasound with Doppler demonstrated patency of the splenic, hepatic, and portal veins. Review of previous imaging revealed that the splenic vein and the superior mesenteric vein (SMV) were occluded prior to the diagnosis of AIP and steroid therapy initiation.

**Outcome:**

Following resolution of hemodynamic instability through fluid resuscitation and blood transfusion, the remainder of his hospital course was uneventful. Subsequent to discontinuation of steroid therapy, he developed near total reocclusion of both the splenic vein and SMV.

**Lesson:**

Early steroid treatment should be considered in patients with uncomplicated AIP to prevent the occlusive vascular complications that are frequently associated with the pathophysiology of this disease process.

## 1 Introduction

Autoimmune pancreatitis (AIP) is a form of chronic pancreatitis now recognized as a distinct clinical entity. AIP has a unique set of serologic, radiologic, and histopathologic features, remaining most well defined by its excellent and rapid clinical response to corticosteroids. The extent of this response is, therefore, contributory to the diagnosis itself in this challenging clinical entity.^[1]^ In this patient, the presence of gastric varices in the absence of splenic vein obstruction prompted additional clinical consideration of the diagnosis in the setting of an atypical presentation. This unexpected constellation of findings provides valuable insight into the effects of corticosteroids on early venous obstruction, a well-recognized component of the natural history of AIP.

## 2 Case presentation

A 50-year-old man presented to the emergency department following 2 days of lightheadedness, abdominal pain, and melena. His distant past medical history was significant for hypertension and transfusion-requiring erosive gastritis. In addition, he reported having been diagnosed with AIP 2 weeks prior at a geographically-proximate academic medical center, where a mass at the head of the pancreas was identified after he presented with abdominal pain, elevated lipase, and hyperglycemia. At that time, he was prescribed prednisone 20 mg PO b.i.d., therapy with which he was compliant. Additional medications prior to admission were diltiazem and tramadol. He has no history of drug or alcohol abuse and denies use of nonsteroidal anti-inflammatory drugs.

On presentation, the patient was in no apparent distress, afebrile, and not hypoxic with a blood pressure of 175/39 mm Hg, a pulse of 140, and a respiratory rate of 18. On physical examination, bowel sounds were normoactive and the abdomen was soft without guarding or rigidity, but significant for marked epigastric tenderness without rebound. There was no palpable splenomegaly, Castell sign was negative and percussion of Traub Space was tympanic. There was no appreciable jaundice on the integument, the sclera, or the oral frenulum. Melena was present on rectal examination. Complete blood count revealed a white blood cell count of 46,000/mL, a hemoglobin of 9.2 mg/dL (baseline 11), and platelets of 96,000/mL. We attributed the leukocytosis to a combination of steroid therapy and stress response to the gastrointestinal bleed. The patient's metabolic panel was significant for a creatinine of 1.8 mg/dL, aspartate aminotransferase of (AST) 40 U/L, alanine aminotransferase of (ALT) 59 U/L, a total bilirubin of 0.7 mg/dL, serum glucose of 300 mg/dL, and a lipase of 31 U/L. IgG4 levels were not drawn as they were not pertinent to the immediate management of the patient's acute gastrointestinal bleed. The patient became acutely hypotensive, at which time an electrocardiogram revealed sinus tachycardia at a rate of 120 beats per minute. Following fluid resuscitation, the hemodynamic compromise resolved. Subsequent hemoglobin measurements revealed a temporal decrement from 9.2 to 7.8 mg/dL. Following initiation of maintenance fluids and a famotidine drip, the patient was admitted to the intensive care unit, where he also received blood transfusions. Due to concerns regarding diagnostic clarity and the veracity of the AIP diagnosis in the setting of gastrointestinal bleeding, the steroid therapy was discontinued.

On hospital day 2, the patient had continued abdominal pain and an additional episode of transfusion-requiring melena, absent vital sign abnormalities. The remainder of laboratory studies were significant for a platelet count of 79,000/mL, a hemoglobin A1c (HbA1c) of 7.0, and a reduction in leukocyte count to 24,500/mL, as expected following discontinuation of steroid therapy. The maintenance fluids and famotidine drip were continued with morphine as needed for abdominal pain.

**Figure 1:**
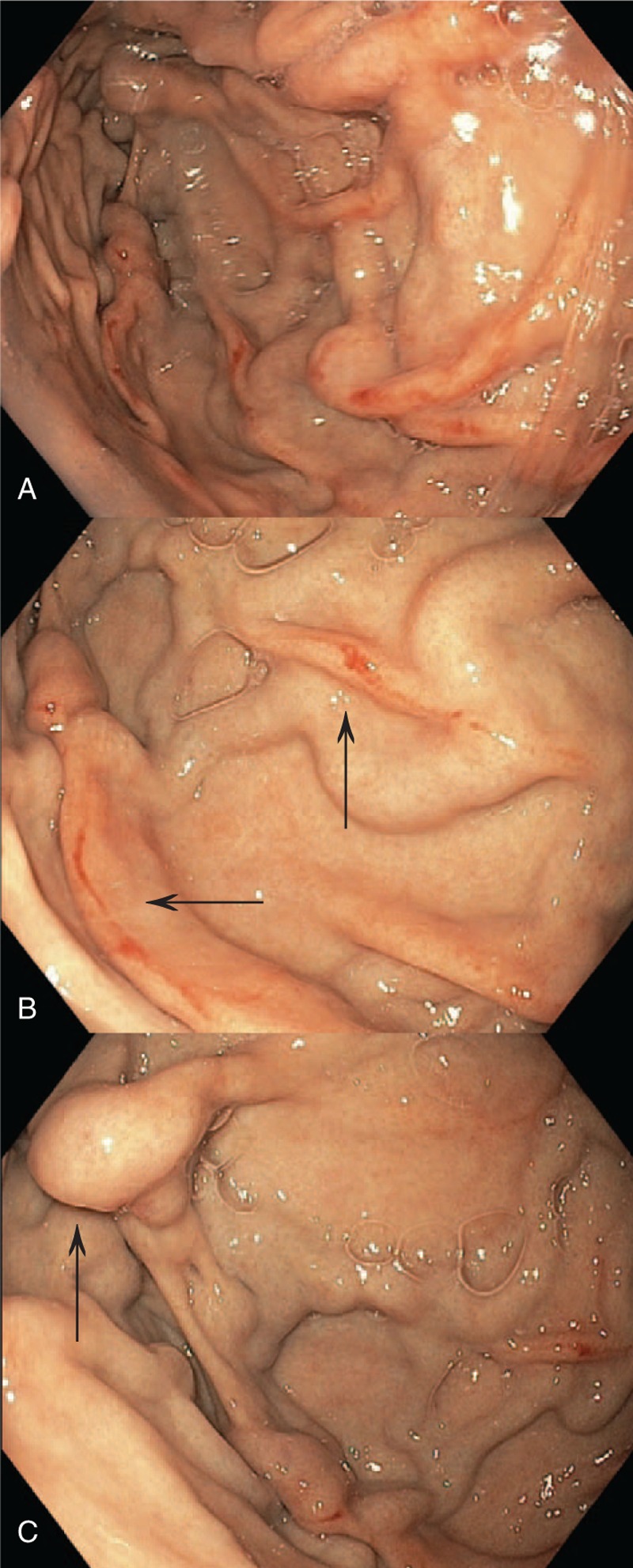
Esophagogastroduodenoscopy shows gastric varices in the fundus. A, Tortuous varices with longitudinal red streaks in the mucosa known as a “red wale sign” or “red wale spots” (arrows), which are associated with an increased risk of variceal bleeding. B, A closer view of torturous linear varices in the fundus with red wale spots (arrows). C, Linear varices and a prominent “bead-shaped” (arrow) varix in the fundus.

On hospital day 3, esophagogastroduodenoscopy (EGD) revealed multiple erosions in the duodenal bulb with surrounding mucosal edema and a 3.5 cm ulcer with an adjacent crater and exudate in the bulb on the anterior wall. Erosive gastritis was also present in the antrum, body, and fundus of the stomach; tissue biopsy was negative for *H Pylori*. Grade 2+ gastric varices were found along the gastric fundus (Fig. 1) and grade 1+ nonbleeding varices were present in the distal esophagus. Abdominal ultrasound with Doppler studies demonstrated moderate splenic enlargement, but patency of the splenic, hepatic, and portal veins.

The patient remained in stable condition throughout hospital days 4 to 7. The remainder of his hospital course was unremarkable with the exception of intermittent melena. He was discharged on hospital day 7, tolerating a full diet. Discharge medications were pantoprazole, prednisone 20 mg PO b.i.d., propranolol, sucralfate, and tramadol as needed for abdominal pain. In the outpatient setting, his care team titrated the dose of propranolol to avert significant heart rate decrement.

## 3 Outpatient course and follow-up

**Figure 2:**
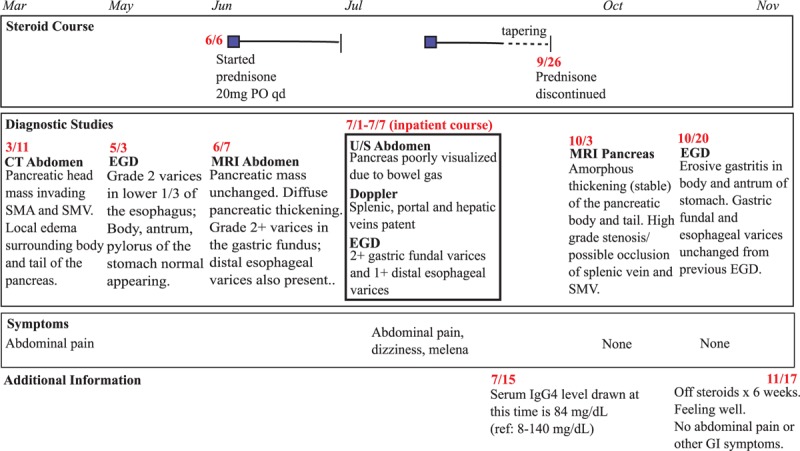
Timeline depicting the temporal relationship of our patient's clinical condition and the steroid therapy administered, including findings on various diagnostic imaging studies.

At outpatient follow-up, the patient reported no complaints. IgG4 levels drawn 1 week after discharge were within normal limits (84 mg/dL; ref: 8–140 mg/dL); hemoglobin was stable at 13.1 mg/dL. His steroid therapy was tapered and discontinued 10 weeks after discharge. One week after discontinuation of steroid therapy, a magnetic resonance imaging (MRI) of the pancreas showed a stable/unchanged amorphous thickening of the body and tail with some reduction in the inflammatory changes visualized in the pancreatic head on a previous MRI. In addition, a 3-month postdischarge MRI done shortly after discontinuation of the corticosteroid revealed high grade stenosis and near complete occlusion of the splenic vein with narrowing of the distal superior mesenteric vein (SMV). At 6-month follow-up, he was asymptomatic despite persistent occlusion of the splenic vein and narrowing of the SMV (see Fig. 2 for temporal clinical course).

## 4 Discussion

AIP is not part of most physicians’ fundamental clinical acumen, given its low epidemiological prevalence. The diagnosis is generally based on a clinical presentation that includes recurrent pancreatitis often complicated by ductal strictures and a pancreatic mass.^[2]^ Although tissue biopsy is the most definitive method of diagnosis, it is supported by clinical history, elevated serum IgG4 levels, imaging that reveals a diffusely enlarged pancreas with featureless borders, and response to corticosteroids. While some cases of AIP resolve spontaneously, induction of remission with corticosteroids achieves rapid clinical improvement in up to 99% of cases and has been shown to improve long-term outcomes and decrease recurrence rates.^[3–5]^

Splenic vein obstruction has been reported to complicate at least 7% to 20% of cases of AIP,^[6–8]^ with gastric varices present in 50% to 75% of such cases.^[6,9]^ One study that compared 46 patients with AIP to matched patients with chronic pancreatitis of alternative etiologies identified splenic vein involvement in 67% of AIP cases, with 39% of those cases having severe stenosis (>70% lumen occluded).^[10]^ Obstruction and thrombosis of the splenic vein occur due to pancreatic inflammation and fibrosis which can damage the vein directly or hinder venous flow through external compression. Such a blockage produces a distinctive left-sided, or “sinistral,” portal hypertension that leads to the development of collateral vasculature commonly involving the gastroepiploic vein and short gastric vessels. In the absence of therapeutic intervention, potential complications of this obstructed vascular flow include progression to congestive splenomegaly and variceal hemorrhage.

Goto et al^[11]^ reported 3 cases of AIP complicated by gastric varices and proposed that the reversibility of varices was related to the presence of splenomegaly at the time of AIP diagnosis. This study highlighted the prospect that corticosteroids appear, observationally, to reduce the development and potential reversibility of gastric varices in patients with AIP. Matsubayashi et al exhibited a markedly greater degree of splenomegaly in AIP patients relative to normal controls and comparators with pancreatitis of other etiologies. They also demonstrated a greater reduction in splenic volume and splenic vein occlusion after corticosteroid therapy among 46 AIP patients relative to patients with chronic pancreatitis of other etiologies.^[10]^ This study also demonstrated that patients treated with steroid therapy achieved a substantial resolution of splenic vein compromise in all cases, with 87% showing complete recanalization. The authors concluded that early corticosteroid use has a direct influence on splenic vein obstruction through a reduction in pancreatic inflammation, leading them to recommend that steroids are a critical therapeutic option when gastric varices are found early in the course of AIP.

Studies based on serial radiographic examinations have shown that early treatment with corticosteroids during the active phase of AIP produces a rapid reduction in pancreatic edema and reversal of pathologic morphological changes in a majority of cases.^[12,13]^ In addition, Hirano et al^[3]^ showed the incidence of “unfavorable events” (including distal bile duct stenosis, pseudocyst enlargement, and extrapancreatic complications) at a mean follow-up of 23 months decreased by more than half when patients were treated with corticosteroids rather than supportive therapy. Similarly, defining relapse as reappearance of symptoms, development of pancreatic or extrapancreatic abnormalities on imaging, and/or marked elevation of serum IgG4 levels, Takuma et al^[5]^ reported relapse among 27% of patients who received standard corticosteroid treatment compared to 45% in patients who did not. These findings support the conclusions of our report and related literature, suggesting that prompt initiation of corticosteroids proactively prevents venoocclusive complications, thus reducing the risk for sinistral portal hypertension and associated vascular complications.

The current literature suggests that steroid therapy is indicated in AIP patients with obstructive jaundice, refractory abdominal pain, diffuse pancreatic enlargement, and symptomatic extrapancreatic lesions, such as hydronephrosis.^[14]^ However the routine use of steroids to induce remission in patients with uncomplicated disease remains controversial.^[3]^ In the patient about whom we report, we assert that transient obstruction of the splenic vein persisted of a duration sufficient to cause at least transient sinistral portal hypertension and subsequent development of gastrosplenic varices. Despite this, steroid therapy was the crucial step in leading to resolution of the obstruction, thereby averting further irreversible complications. We cannot know with certainty whether early steroid therapy could have prevented the formation of gastrosplenic varices in this case, but we conjecture a potential benefit of this intervention. The details of this case report closely parallel those of the 3 patients reported by Goto et al and support their suggestion that the early presence of splenomegaly suggests that associated gastrosplenic varices may not resolve in the absence of therapy. These findings are also substantiated by considerable recent literature advocating early corticosteroid use for patients with uncomplicated AIP to modify the natural history of the disease process.^[3,4,10,11,15]^ We believe that early intervention with steroids and noninvasive radiological screening for venous obstruction (contrast enhanced CT or abdominal ultrasound) should be given strong consideration in patients with uncomplicated AIP.

## 5 Conclusion

We report an unusual case of autoimmune pancreatitis complicated by gastrosplenic varices with perspectives based on the evidence available suggesting a disease-modifying benefit of steroid treatment in a subset of patients not conventionally thought to benefit from corticosteroids. In our patient, gastric varices were discovered on EGD after 2 weeks of steroid treatment without concurrent splenic vein obstruction. A substantial amount of clinical, radiological, and pharmacological evidence supports our belief that early steroid treatment directly reduces the risk of irreversible complications from splenic vein obstruction. Early corticosteroid treatment has important short and long-term benefits among a large proportion of AIP patients to reduce the possibility of permanent complications related to splenic vein obstruction and sinistral portal hypertension.

## Acknowledgments

The authors thank Dr Julie Bauml (radiology) and Dr Magdy El-Din (internal medicine PGY-2) for their clinical insights.

## Author contributions

**Conceptualization:** Lindsay Juarez, Gregory Ruhnke.

**Formal analysis:** Lindsay Juarez, Gregory Ruhnke.

**Validation:** Roop Gupta, Gregory Ruhnke.

**Writing – original draft:** Lindsay Juarez.

**Writing – review & editing:** Lindsay Juarez, Roop Gupta, Gregory Ruhnke.
